# Synthesis, Structure of Nitrogen-Containing Phosphinogold(I) Ferrocenes. *In vitro* Activity against Bladder and Colon Carcinoma Cell Lines

**DOI:** 10.1155/MBD.1995.311

**Published:** 1995

**Authors:** Manuella Viotte, Bernard Gautheron, Marek M. Kubicki, Ilya E. Nifant'ev, Simon P. Fricker

**Affiliations:** 1 Laboratoire de Synthèse et d'Electrosynthèse Organométalliques URA CNRS 1685 Université de Bourgogne BP 138 Dijon Cedex F-21004 France; 2 Organic Division Department of Chemistry Moscow University Moscow 119899 Russia; 3 Johnson Matthey Technology Centre Blount’s Court Sonning Common Reading RG4 9NH United Kingdom

## Abstract

The gold salt [(tht)AuCl] was reacted with [1-N,N-dimethylaminométhyl-2-diphenylphosphino]ferrocene (1) forming the bimetallic derivative 4. The reaction of methyl iodide and tetramethylammonium bromide on the chloride 4 produced the ammonium salt 5 and the bromide 6 respectively. New aminophosphines 2 and 3, which represent two of the rare phosphorylated metallocenes containing P(III)-N bond have also been coordinated to gold(I) to form 7 and 8. The presence of the ethoxy group in 7 provides evidence for the lability of one nitrogen-phosphorus bond. The X-ray structure of compounds 4 and 7 have been established. Both crystallize in space group P2_1_/c, monoclinic, with a = 11.095(2) Å, b = 12.030(3) Å, c = 17.763(4) Å, β= 94.02(2)∘, Z = 4 for 4 and a = 14.863(3) Å, b = 8.036(5)Å, c = 18.062(5)Å, β =101.64(1)°, Z = 4 for 7. ^197^Au  Mössbauer data are in good agreement with those for other linear P-Au-Cl containing complexes. The compounds were evaluated for *in vitro* anti-tumour activity against two human tumours. Differential cytotoxicity was observed with activity comparable to cisplatin, with the exception of one compound which was significantly more cytotoxic.


### SYNTHESIS, STRUCTURE OF NITROGEN-CONTAINING PHOSPHINOGOLD(I) FERROCENES. *IN VITRO* ACTIVITY AGAINST BLADDER AND COLON CARCINOMA CELL LINES

Manuella Viotte[1], Bernard Gautheron*[1], Marek M. Kubicki[1], Ilya E. Nifant'ev[2] and Simon P. Fricker[3]

[1] Laboratoire de Synthèse et d'Electrosynthèse Organométalliques, URA CNRS 1685, Université de Bourgogne, BP 138, F-21004 Dijon Cedex, France

[2] Organic Division, Department of Chemistry, Moscow University, Moscow 119899, Russia

[3] Johnson Matthey Technology Centre, Blount's Court, Sonning Common, Reading RG4 9NH, U.K.

### ABSTRACT

The gold salt [(tht)AuCl] was reacted with [1-N,N-dimethylaminométhyl-2-diphenylphosphino]ferrocene (**1**) forming the bimetallic derivative **4**. The reaction of methyl iodide and tetramethylammonium bromide on the chloride **4** produced the ammonium salt **5** and the bromide **6** respectively. New aminophosphines **2** and **3**, which represent two of the rare phosphorylated metallocenes containing P(III)-N bond have also been coordinated to gold(I) to form **7** and **8**. The presence of the ethoxy group in **7** provides evidence for the lability of one nitrogen-phosphorus bond. The X-ray structure of compounds **4** and **7** have been established. Both crystallize in space group $P2_1/c$, monoclinic, with a = 11.095(2) Å, b = 12.030(3) Å, c = 17.763(4) Å, β = 94.02(2)°, Z = 4 for **4** and a = 14.863(3) Å, b = 8.036(5) Å, c = 18.062(5) Å, β = 101.64(1)°, Z = 4 for **7**. $^{197}$Au Mössbauer data are in good agreement with those for other linear P-Au-Cl containing complexes. The compounds were evaluated for *in vitro* anti-tumour activity against two human tumours. Differential cytotoxicity was observed with activity comparable to cisplatin, with the exception of one compound which was significantly more cytotoxic.

**Keywords**: heteropolymetallic complexes (Fe, Au), antitumoural activity, X-ray structure, NMR and $^{197}$Au Mössbauer spectroscopies

---

* Author to whom correspondence should be addressed.

### INTRODUCTION

The antitumour activity of diphenylphosphine complexes is of recent interest and it has been proved that the coordination of the diphosphine to gold(I) enhances the cytotoxicity.[1,2] Moreover the intimate association of two therapeutic metals in the same molecule could give the possibility of a synergism between them. With this objective in mind, we have prepared gold(I) derivatives of compounds similar to $[FcCH_2NHMe_2]^+[Cl]^-$ (Fc = $\eta^5$-$C_5H_5FeC_5H_4$) which itself exhibits significant antitumour activity[3]. We report here the synthesis and comparative *in vitro* cytotoxicity of the (diphenylphosphino)chlorogold(I) complex obtained from $[FcCH_2NMe_2]$), the corresponding iodide salt, the bromide derivative, new ferrocenylphosphines and their gold(I) complexes.

### MATERIALS AND METHODS

All reactions were conducted under an atmosphere of pure dry argon. Solvents were dried and deoxygenated over sodium/benzophenone ketyl and distilled immediately before use. $CH_2Cl_2$ stabilized with 0.3 % ethanol was purchased from SDS Company. Transfers were carried out via syringes or cannulas.

Microanalyses were performed by the Microanalysis Centre in UMIST (Manchester, U.K.). Melting points were determined with a Kofler apparatus without correction. $^1H$, $^{13}C\{^1H\}$, $^{19}F$ and $^{31}P$ nuclear magnetic resonance spectra were recorded in $CDCl_3$ with a Bruker AC 200 spectrometer operating at 200.0, 50.3, 187.8 and 81.0 MHz respectively. Chemical shifts are reported in $\delta$ units, parts per million downfield from internal tetramethylsilane for $^1H$, $^{13}C\{^1H\}$ and from external $H_3PO_4$ for $^{31}P$.

The Mössbauer spectra were obtained in the Technische Universität München (Garching, Germany). The $^{197}Pt$ activity feeding the 77.3 keV Mössbauer transition was produced by irradiation of enriched $^{196}Pt$ metal. Both source and absorber were kept at 4.2 K, a sinusoïdal velocity wave form and an intrinsic Ge detector were used. A sample of the absorber with of ca. 50 mg(Au).cm$^{-2}$ was used, spectra were fitted to Lorentzian lines.

### Ligand Synthesis

$[Na(AuCl_4), 2H_20]$ was a generous gift from Johnson Matthey Technology Centre. (1-N,N-dimethylaminomethyl-2-diphenylphosphino)ferrocene (**1**),[4, 5, 6] [(tht)AuCl],[7] $[ClP(NMe_2)_2]$ and $[H_2CN(CH_3)PN(CH_3)CH_2]Cl$[8] were prepared by literature methods.

**[1-N,N-dimethylaminométhyl-2-bis(dimethylamino)phosphino]ferrocene (2)**

A solution of BuLi in hexane 1.45 M (4.30 ml, 6.17 mmol) was added dropwise to a stirred suspension of N,N-dimethylaminomethylferrocene (1.50 g, 6.17 mmol) in diethylether (5 ml) at 0 °C. Stirring was maintained until the precipitate of *o*-lithioamine appeared. This monolithioamine was reacted with [ClP(NMe$_2$)$_2$] (0.95 g, 6.17 mmol) and the resultant mixture was heated under reflux for 1 hr. The white solid (LiCl) was filtered off and the solvent was taken off. The oil obtained was recrystallized from pentane at -78 °C yielding 0.60 g (27 %) of a pure brown oil (at room temperature). Anal. Calcd. for $C_{17}H_{28}FeN_3P$: C, 56.5; H, 7.8; N, 11.6; Fe, 15.4. Found: C, 56.6; H, 7.6; N, 11.3; Fe, 15.1. $^{1}$**H NMR** ($C_6D_6$): 2.15 (s, 6 H, CH$_2$N*Me$_2$*), 2.63, 2.72 (d, 6 H, $^{3}J_{H-P}$ = 9 Hz, PNMe$_2$), 3.16 (d, 1 H, $^{2}J_{H-H}$ = 13 Hz, CH$_2$), 3.56 (dd, 1 H, $^{2}J_{H-H}$ = 13 Hz, $^{4}J_{H-P}$ = 2 Hz, CH$_2$),4.10 (s, 5 H, Cp), 4.08- 4.13, 4.23- 4.26, 4.32- 4.37 (m, 1 H, Cp). $^{31}$**P NMR**: 93.4 (s). $^{13}$**C NMR** ($C_6D_6$): 41.3, 42.1 (d, $^{2}J_{C-P}$ = 17 Hz, PNMe$_2$), 45.7 (s, CH$_2$N*Me$_2$*), 58.8 (d, $^{3}J_{C-P}$ = 10 Hz, CH$_2$), 68.2, 70.4, 71.1, 72.8 (s, Cp), 69.6 (d, $^{1}J_{C-P}$ = 79 Hz, C *ipso*), 88.2 (d, $^{2}J_{C-P}$ = 19 Hz, *C*-CH2).

**[1-N,N-dimethylaminomethyl-2-((N,N-dimethyl)-2,5-diaza-1-phospholanyl)]-ferrocene (3)**

A solution of BuLi in hexane 2.35 M (2.40 ml, 5.58 mmol) was added dropwise to a stirred suspension of N,N-dimethylaminomethylferrocene (1.36 g, 5.58 mmol) in diethylether (5 ml) at 0 °C. Stirring was maintained until the precipitate of *o*-lithioamine appeared. [H$_2$CN(CH$_3$)PN(CH$_3$)CH$_2$]Cl (0.85 g, 5.58 mmol) was added to the lithioamine and the resultant mixture was heated under reflux for 1 hr. The white solid (LiCl) was filtered off and the solvent was eliminated under vaccum. The solid obtained was recrystallized from pentane yielding 0.60 g (27 %) of pure orange crystals.Mp: 84 °C. Anal. Calcd. for $C_{17}H_{26}FeN_3P$: C, 56.8; H, 7.3; N, 11.7; Fe, 15.5. Found: C, 56.9; H, 8.0; N, 11.2; Fe, 14.6. $^{1}$**H NMR** ($C_6D_6$): 2.10 (s, 6 H, CH$_2$N*Me$_2$*), 2.57, 2.81 (d, 3 H, $^{3}J_{H-P}$ = 14 Hz, PNMe), 2.66-2.93 (m, 3 H, PNCH$_2$), 2.79 (d, 1 H, $^{2}J_{H-H}$ = 12 Hz, *CH$_2$*NMe$_2$), 3.11-3.15 (m, 1 H, PNCH$_2$), 3.98 (dd, 1 H, $^{2}J_{H-H}$ = 12 Hz, $^{4}J_{H-P}$ = 2 Hz, *CH$_2$*NMe$_2$), 4.01- 4.04, 4.07- 4.11, 4.13- 4.17 (m, 1 H, Cp), 4.12 (s, 5 H, Cp), $^{31}$**P NMR**: 100.2 (s). $^{13}$**C NMR** ($C_6D_6$): 36.0, 39.9 (d, $^{2}J_{C-P}$ = 15 Hz, PNMe), 45.2 (s, CH$_2$N*Me$_2$*), 54.2, 55.2 (d, $^{2}J_{C-P}$ = 9 Hz, PNCH$_2$), 58.6 (d, $^{3}J_{C-P}$ = 8 Hz, *CH$_2$*NMe$_2$), 68.5, 69.2, 71.5, 73.4 (s, Cp), 82.3 (d, $^{1}J_{C-P}$ = 54 Hz, C *ipso*), 90.2 (d, $^{2}J_{C-P}$ = 21 Hz, *C*-CH2).

**Synthesis of Gold(I) Complexes**

**(1-N,N-dimethylaminomethyl-2-chlorogold(I)diphenylphosphino)ferrocene (4)**

A solution of **1** (0.163 g, 0.382 mmol) in methylene chloride (20 ml) was added to a solution of [(tht)AuCl] (0.123 g, 0.382 mmol) in the same solvent (20 ml) at room temperature. Stirring was maintained for two hours and the solvent was then evaporated. The orange residue was recrystallized from toluene. Yield (0.186 g, 73 %). Mp: 206 °C. Anal. Calcd. for $C_{25}H_{26}AuClFeNP$: C, 45.5; H, 4.0; N, 2.1; Fe, 8.5. Found: C, 45.5; H, 3.9; N, 2.1; Fe, 8.0. $^{1}$H **NMR** ($C_6D_6$): 1.86 (s, 6 H, Me), 2.70, 4.66 (d, 1 H, $^{2}J_{H\text{-}H}$ = 13 Hz, $CH_2$), 3.55-3.59, 3.83-3.86, 4.08-4.11 (m, 1 H, Cp), 3.99 (s, 5 H, Cp), 6.81-7.04 (m, 6 H, *m*-H, *p*-H), 7.16-7.31, 7.54-7.69 (m, 2 H, *o*-H), $^{31}$P **NMR**: 20.2 (s). $^{13}$C **NMR** ($CDCl_3$): 44.5 (s, Me), 58.7 (s, $CH_2$), 69.4 (d, $^{1}J_{C\text{-}P}$ = 73 Hz, C *ipso*), 69.8, 73.7, 75.2 (d, $J_{C\text{-}P}$ = 8, 5, 8 Hz, Cp), 70.9 (s, Cp), 90.2 (d, $^{2}J_{C\text{-}P}$ = 14 Hz, *C*-CH2), 128.4, 128.7 (d, $J_{C\text{-}P}$ = 12 Hz), 130.3, 131.7 (d, $^{1}J_{C\text{-}P}$ = 65 Hz, C *ipso*), 130.9, 131.7 (s, *p*-C), 133.1, 134.8 (d, $J_{C\text{-}P}$ = 14 Hz). $^{197}$Au **Mössbauer** ($mm.s^{-1}$): QS = 7.71, IS = 4.19.

**Ammonium salt from (1-N,N-dimethylaminomethyl-2-iodogold(I)-diphenylphosphino)ferrocene(5)**

Two equivalents of methyl iodide was added to a solution of **4** (0.106 g, 0.160 mmol) in acetonitrile (20 ml) at room temperature. On addition of diethylether an orange powder was formed. This powder does not melt but darkened and decomposed from 202 °C. Yield (0.102 g, 71 %). Anal. Calcd. for $C_{26}H_{29}AuFeI_2NP$: C, 35.0; H, 3.3; N, 1.6; I, 28.4. Found: C, 35.6; H, 3.2; N, 2.2; I, 27.0. $^{1}$H **NMR** ($CH_3CN$): 2.16 (s, 6 H, Me), 4.84, 5.70 (d, 1 H, $^{2}J_{H\text{-}H}$ = 14 Hz, $CH_2$), 3.98-4.06, 4.90-4.95, 5.07-5.12 (m, 1 H, Cp), 4.26 (s, 5 H, Cp), 7.45-7.73 (m, 8 H, *m*-H, *p*-H, *o*-H), 7.83-7.99 (m, 2 H, *o*-H), $^{31}$P **NMR**: 29.9 (s).

**(1-N,N-dimethylaminomethyl-2-bromogold(I)diphenylphosphino)-ferrocene (6)**

A solution of $Et_4NBr$ (0.062 g, 0.296 mmol) in ethanol (20 ml) was added to a slurry of **4** (0.098 g, 0.148 mmol) in the same solvent (20 ml) at -60 °C. Stirring was maintained for a few hours during which the solution warmed slowly to room temperature. The orange solid formed was isolated and washed with ethanol and diethylether. It did not melt but darkened and decomposed from 206 °C. Yield (0.085 g, 82 %). Anal. Calcd. for $C_{25}H_{26}AuBrFeNP$: C, 42.6; H, 3.7; N, 2.0; Br, 11.4. Found: C, 42.7; H, 3.7; N, 2.1; Fe, 11.6. $^{1}$H **NMR** ($C_6D_6$): 1.89 (s, 6 H, Me), 2.72, 4.71 (d, 1 H, $^{2}J_{H\text{-}H}$ = 13 Hz, $CH_2$), 3.60-3.64, 3.88-3.92, 4.10-4.14 (m, 1 H, Cp), 4.06 (s, 5 H, Cp), 6.85-7.10 (m, 6 H, *m*-H, *p*-H), 7.22-7.35, 7.61-7.73 (m, 2 H, *o*-H), $^{31}$P **NMR**: 25.8 (s).

**[1-N,N-dimethylaminomethyl-2-(chlorogold(I)dimethylaminoethoxy)-phosphino]ferrocene (7)**

This compound was prepared from **2** (0.608 g, 1.680 mmol) and [(tht)AuCl] (0.540 g, 1.608 mmol) by a procedure similar to that given for **4**. The orange solid was recrystallized from pentane. Yield (0.180 g, 20 %). Mp: 116 °C. Anal. Calcd. for $C_{17}H_{27}AuClFeN_2OP$: C, 34.3; H, 4.6; N, 4.7; Fe, 9.4. Found: C, 34.7; H, 4.6; N, 4.8; Fe, 8.9. $^{1}$**H NMR** ($C_6D_6$): 1.00 (t, 3 H, $OCH_2$*$CH_3$*), 2.01 (s, 6 H, $CH_2N$*$Me_2$*), 2.31 (d, 6 H, $^{3}J_{H-P}$ = 11 Hz, $PNMe_2$), 2.67 (d, 1 H, $^{2}J_{H-H}$ = 13 Hz, *$CH_2$*$NMe_2$), 3.42-3.75 (m, 2 H, O*$CH_2$*$CH_3$), 4.47 (d, 1 H, $^{2}J_{H-H}$ = 13 Hz, *$CH_2$*$NMe_2$), 3.89- 3.94, 3.97- 4.04, 4.29- 4.33 (m, 1 H, Cp), 4.18 (s, 5 H, Cp). $^{31}$**P NMR**: 115.7 (s). $^{13}$**C NMR** ($C_6D_6$): 16.3 (d, $^{3}J_{C-P}$ = 8.4 Hz, $OCH_2$*$CH_3$*), 38.1 (d, $^{2}J_{C-P}$ = 9 Hz, $PNMe_2$), 44.8 (s, $CH_2N$*$Me_2$*), 58.9 (d, $^{3}J_{C-P}$ = 2 Hz, $CH_2NMe_2$), 64.2 (s, $OCH_2CH_3$), 68.8, 72.4, 74.4 (d, $J_{C-P}$ = 8, 3, 9 Hz, Cp), 71.0 (s, Cp), 72.7 (d, $^{1}J_{C-P}$ = 107 Hz, C *ipso*), 89.8 (d, $^{2}J_{C-P}$ = 21 Hz, *C*-CH2). $^{197}$**Au Mössbauer** ($mm.s^{-1}$): QS = 7.74, IS = 4.46.

**[1-N,N-dimethylaminomethyl-2-(N,N-dimethyl-2,5-diaza-chlorogold(I)-1-phospholanyl)]ferrocene (8)**

This compound was prepared from **3** (0.494 g, 1.374 mmol) and [(tht)AuCl] (0.441 g, 1.374 mmol) by a procedure similar to that given for **4**. The orange solid was recrystallized from toluene/pentane. Yield (0.183 g, 22 %). Mp: 150 °C. Anal. Calcd. for $C_{17}H_{26}AuClFeN_3P$: C, 34.5; H, 4.4; N, 7.1; Fe, 9.4. Found: C, 34.7; H, 4.4; N, 7.1; Fe, 9.1. $^{1}$**H NMR** ($C_6D_6$): 2.12 (s, 6 H, $CH_2N$*$Me_2$*), 2.40, 2.43 (d, 3 H, $^{3}J_{H-P}$ = 14 Hz, PNMe), 2.36-2.52 (m, 3 H, $PNCH_2$), 2.53 (d, 1 H, $^{2}J_{H-H}$ = 13 Hz, *$CH_2$*$NMe_2$), 2.67-2.74 (m, 1H, $PNCH_2$), 4.60 (d, 1 H, $^{2}J_{H-H}$ = 12 Hz, *$CH_2$*$NMe_2$), 3.84- 3.89, 3.93-3.95, 4.00-4.04 (m, 1 H, Cp), 4.14 (s, 5 H, Cp), $^{31}$**P NMR**: 103.2 (s). $^{13}$**C NMR** ($C_6D_6$): 34.3, 36.3 (d, $^{2}J_{C-P}$ = 11 Hz, PNMe), 44.7 (s, $CH_2N$*$Me_2$*), 51.5, 52.0 (s, $PNCH_2$), 58.8 (s, *$CH_2$*$NMe_2$), 69.4, 72.4, 75.7 (d, $J_{C-P}$ = 8, 10, 7 Hz, Cp), 70.2 (s, Cp), 77.3 (d, $^{1}J_{C-P}$ = 62 Hz, C *ipso*), 89.1 (d, $^{2}J_{C-P}$ = 10 Hz, *C*-CH2). $^{197}$**Au Mössbauer** ($mm.s^{-1}$): QS = 7.47, IS = 4.48.

**Crystallographic Studies**

Orange crystals of **4** and **7** were grown from toluene and pentane solutions, respectively. They were mounted on an Enraf-Nonius CAD4 diffractometer. The pertinent crystallographic data are given in Table I. The unit cells were determined from 25 randomly selected reflections ($8<\theta<18$). The intensity data were collected at room temperature with $\omega/2\theta$ scan up to $\theta_{max}$ = 25°. The Enraf-Nonius Molen package was used for preliminary treatement of data. The intensities were corrected for standard Lorentz and polarisation effects and empirical absorption correction applied for both crystals. The structures were solved and refined by conventional three-dimensional Patterson, difference Fourier, and full-matrix least-squares methods by using the SHELX76 package. All nonhydrogen atoms in both structures were refined with anisotropic temperature factors. The hydrogen atoms were placed in calculated positions and were given riding on the atoms bearing them. The highest peaks in the final difference map are in close proximity to the gold atoms. Final atomic coordinates are given in Tables II and III. Tables of hydrogen atom positions, anisotropic temperature factors and observed and calculated structure factors may be obtained from the authors (MMK)

**Table I.** Crystallographic Data for $[Fc(CH_2NMe_2)(PPh_2AuCl)]$ (**4**) and $[Fc(CH_2NMe_2)(P(NMe_2)(OCH_2CH_3)(AuCl)]$ (**7**)

| | | |
|---|---|---|
| chem. formula | $C_{25}H_{26}AuClFeNP$ | $C_{17}H_{27}AuClFeN_2OP$ |
| mol wt | 659.73 | 594.66 |
| color | yellow | yellow |
| dimensions | 0.35 x 0.25 x 0.08 | 0.15 x 0.10 x 0.10 |
| space group | $P2_1/c$ (No.14) | $P2_1/c$ (No. 14) |
| a Å | 11.095(2) | 14.863(3) |
| b Å | 12.030(3) | 8.036(5) |
| c Å | 17.763(4) | 18.062(5) |
| β deg | 94.02(2) | 101.64(1) |
| V $Å^3$ | 2365.1 | 2112.9 |
| Z | 4 | 4 |
| $d_{calc}$ $g.cm^{-3}$ | 1.85 | 1.87 |
| F(000) | 1280 | 1152 |
| λ (Mo Kα) Å | 0.73071 | 0.73071 |
| μ (Mo Kα) $cm^{-1}$ | 69.92 | 78.20 |
| temperature, K | 291 | 291 |
| scan type | ϖ-2θ | ϖ-2θ |
| θ range, deg | 2 - 25 | 2 - 25 |
| linear decay, % | -5.3 (corrected) | -4.0 (corrected) |
| tot. no. of refls | 4835 | 4142 |
| unique data $I > 3\sigma(I)$ | 3149 | 2354 |
| no. of variables | 271 | 217 |
| absorption correction (Ψ scan) | 71.520 - 99.969 | 67.553-99.814 |
| R | 0.023 | 0.037 |
| Rw | 0.027 | 0.039 |
| GOF | 0.815 | 1.188 |
| weighting scheme | | |
| $w = 1/(\sigma^2(F) + g F^2)$, g | 0.00138 | 0.00077 |
| residual density, e $Å^{-3}$ | 0.71 | 1.1 |

### Biological Studies

Evaluation of potential anti-tumour activity was conducted against two human tumour cell lines, the SW620 colon carcinoma[9] and the HT1376 bladder carcinoma[10] The cell lines were obtained from the ECACC. The cells were seeded onto 96-well microtitre plates at a concentration

**Table II.** Atomic Coordinates for $[Fc(CH_2NMe_2)(PPh_2AuCl)]$ (**4**)

| Atom | x | y | z | $B_{eq}(Å^3)$ |
|---|---|---|---|---|
| Au | 0.23768(2) | 0.39337(2) | 0.35602(1) | 2.937(6) |
| Fe | 0.34965(6) | 0.04916(7) | 0.36027(4) | 2.36(2) |
| Cl | 0.1998 (1) | 0.4997 (1) | 0.2511 (1) | 4.48(4) |
| P | 0.2794 (1) | 0.2886 (1) | 0.4576 (1) | 2.73(3) |
| N | 0.0045 (4) | 0.2062 (4) | 0.3917 (2) | 3.54(11) |
| C1 | 0.2729 (4) | 0.1421 (4) | 0.4396 (3) | 2.84(11) |
| C2 | 0.1832 (4) | 0.0844 (4) | 0.3948 (3) | 2.98(12) |
| C3 | 0.2073 (5) | -0.0312 (4) | 0.4029 (3) | 3.83(14) |
| C4 | 0.3132 (5) | -0.0454 (4) | 0.4522 (3) | 3.75(14) |
| C5 | 0.3549 (4) | 0.0602 (4) | 0.4753 (3) | 3.25(13) |
| C6 | 0.0816 (4) | 0.1361 (5) | 0.3465 (3) | 3.26(13) |
| C7 | -0.0766 (5) | 0.2724 (5) | 0.3436 (3) | 4.81(17) |
| C8 | -0.0617 (5) | 0.1393 (6) | 0.4432 (4) | 4.70(17) |
| C11 | 0.4194 (8) | 0.1493 (6) | 0.2823 (4) | 6.11(23) |
| C12 | 0.3417 (7) | 0.0726 (8) | 0.2465 (4) | 6.74(25) |
| C13 | 0.3855 (8) | -0.0334 (7) | 0.2647 (4) | 6.71(26) |
| C14 | 0.4902 (8) | -0.0220 (7) | 0.3097 (5) | 6.68(25) |
| C15 | 0.5130 (7) | 0.0902 (9) | 0.3210 (5) | 7.49(31) |
| C21 | 0.1811 (4) | 0.3072 (4) | 0.5339 (3) | 3.05(12) |
| C22 | 0.1218 (5) | 0.4083 (4) | 0.5415 (3) | 3.72(14) |
| C23 | 0.0438 (5) | 0.4230 (5) | 0.5974 (3) | 4.39(16) |
| C24 | 0.0197 (5) | 0.3358 (6) | 0.6458 (3) | 4.45(16) |
| C25 | 0.0798 (5) | 0.2366 (6) | 0.6392 (3) | 4.52(16) |
| C26 | 0.1598 (5) | 0.2217 (4) | 0.5847 (3) | 3.56(13) |
| C31 | 0.4325 (4) | 0.3165 (4) | 0.4981 (3) | 3.05(12) |
| C32 | 0.4643 (5) | 0.3123 (5) | 0.5750 (3) | 4.09(15) |
| C33 | 0.5796 (6) | 0.3346 (5) | 0.6026 (4) | 5.07(18) |
| C34 | 0.6668 (6) | 0.3588 (5) | 0.5545 (4) | 5.08(19) |
| C35 | 0.6376 (5) | 0.3651 (6) | 0.4794 (4) | 5.27(19) |
| C36 | 0.5202 (5) | 0.3450 (6) | 0.4494 (3) | 4.69(17) |

$B_{eq}$ values are defined as: $8/3\pi^2(U11 + U22 + U33)$.

The sulforhodamine B(SRB) assay was performed as previously described[11]. The cells were fixed with 200 μl 10 % trichloroacetic acid for 30 minutes at 4°C, washed five times in tap water and stained with 100 μl 0.1 % SRB in 1 % acetic acid for 15 minutes. The cells were washed four times with 1 % acetic acid and air dried. The stain was solubilised in 200 μl 10 mM unbuffered Tris base. Absorbance was measured at 540 nm using a microtitre plate reader.

**Table III.** Atomic Coordinates values for
$[Fc(CH_2NMe_2)(P(NMe_2)(OCH_2CH_3)(AuCl)]$ (**7**)

| Atom | x | y | z | Beq(Å²) |
|---|---|---|---|---|
| Au | 0.25436 (6) | 0.52238 (9) | -0.03566 (4) | 5.05(1) |
| Fe | 0.1558 (2) | 0.5592 (3) | 0.1762 (1) | 5.48(4 |
| P | 0.2432 (4) | 0.7451 (5) | 0.0347 (3) | 4.80(6 |
| Cl | 0.2670 (4) | 0.2951 (7) | -0.1105 (3) | 7.99(9 |
| O | 0.1479 (8) | 0.843 (2) | 0.0095 (7) | 5.9(2) |
| N1 | 0.436 (1) | 0.563 (2) | 0.1366 (9) | 6.3(2) |
| N2 | 0.325 (1) | 0.881 (2) | 0.0320 (8) | 6.0(2) |
| C1 | 0.086 (2) | 0.417 (3) | 0.092 (1) | 9.4(5) |
| C2 | 0.024 (2) | 0.518 (3) | 0.120 (1) | 9.9(5) |
| C3 | 0.033 (1) | 0.476 (4) | 0.198 (2) | 8.7(5) |
| C4 | 0.100 (2) | 0.354 (3) | 0.219 (1) | 8.2(4) |
| C5 | 0.132 (2) | 0.315 (3) | 0.151 (2) | 8.9(5) |
| C6 | 0.238 (1) | 0.711 (2) | 0.1304 (9) | 5.1(2) |
| C7 | 0.292 (1) | 0.605 (2) | 0.183 (1) | 5.5(3) |
| C8 | 0.269 (1) | 0.622 (3) | 0.257 (1) | 6.7(3) |
| C9 | 0.202 (1) | 0.754 (2) | 0.251 (1) | 7.3(3) |
| C10 | 0.182 (1) | 0.807 (2) | 0.1749 (9) | 5.9(3) |
| C11 | 0.360 (1) | 0.468 (2) | 0.162 (1) | 5.5(2) |
| C12 | 0.493 (1) | 0.436 (3) | 0.107 (1) | 8.0(4) |
| C13 | 0.492 (2) | 0.663 (3) | 0.200 (1) | 9.7(4) |
| C14 | 0.396 (1) | 0.855 (3) | -0.013 (1) | 7.8(4) |
| C15 | 0.336 (2) | 1.033 (3) | 0.079 (1) | 8.7(4) |
| C16 | 0.122 (2) | 0.890 (3) | -0.073 (1) | 7.1(3) |
| C17 | 0.057 (2) | 1.029 (4) | -0.079 (1) | 10.2(5) |

Anisotropically refined atoms are given in the form of the isotropic equivalent displacement parameter defined as: (4/3) * [a2*B(1,1) + b2*B(2,2) + c2*B(3,3) + ab(cos γ)*B(1,2) + ac(cos β)*B(1,3) + bc(cos α)*B(2,3)]

### RESULTS AND DISCUSSION

### Synthetic Studies

Compounds **1**, **2** and **3** contain both "soft" (P) and "hard" (N) ligands. It is well known that the former site is normally prefered for coordination by a "soft" metal like gold(I). This has been verified when the gold salt [(tht)AuCl] was reacted with [1-N,N-dimethylaminométhyl-2-diphenylphosphino]ferrocene (**1**) forming the bimetallic derivative **4** (Scheme 1)

**Scheme 1.**

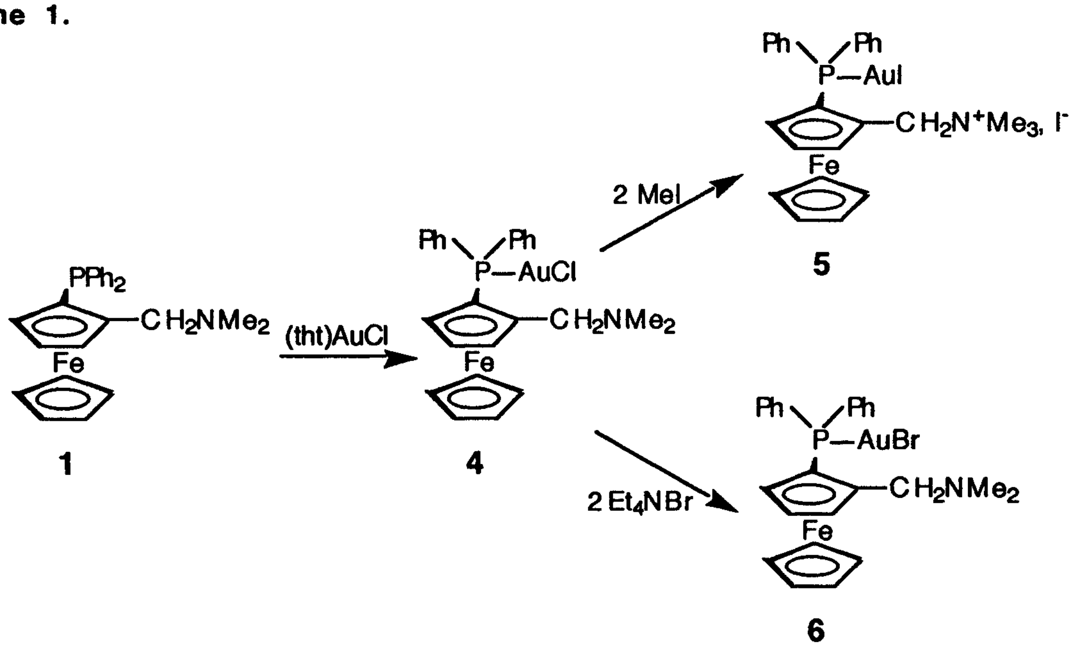

Regarding the positive antitumoral response of **1** before coordination and the inversion in selectivity after coordination with gold (see after), we have decided to synthesize other N- and P-containing derivatives by two different approaches consisting of modification of (1-N,N-dimethylaminomethyl-2-chlorogold(I)diphenylphosphino)ferrocene (**4**) or by using new starting phosphines.

Then two new complexes were synthesized from **4**. The reaction of two equivalents of methyl iodide with **4** produced the corresponding salt (**5**) (the substitution of the chlorine atom by iodine was observed). The bromide **6** was obtained by reacting tetraethylammonium bromide with the chloride **4** (Scheme 1).

The second approach (Scheme 2) allowed the two metallophosphines **2** and **3** to be synthesized by reacting (N,N-dimethylaminomethyl)ferrocene with BuLi and [ClP(NMe$_2$)$_2$] for **2** or BuLi and [H$_2$CN(CH$_3$)PN(CH$_3$)CH$_2$]Cl for **3**. They represent two of the rare phosphorylated metallocenes containing P(III)-N bond[12] which can be coordinated to gold. The unexpected presence of the ethoxy group in **7** is due to the ethanol (0.3 %) used as stabilizer in the commercial CH$_2$Cl$_2$ and the transformation shows evidence for the lability of one of the nitrogen-phosphorus bonds. The $^1$H NMR spectrum in particular exhibits one triplet at 1 ppm (OCH$_2$*CH$_3$*) and one large multiplet (3.42-3.75 ppm) corresponding to the two diastereotopic protons of O*CH$_2$*CH$_3$

multicoupled to each other, with the phosphorus atom and methyl protons. We have not tried to discard ethanol from methylene chloride.

The $^{1}H$ and $^{13}C$ NMR spectra of each compound reflects the diastereotopy of the $PNMe_2$ methyl substituents, the phenyl protons, and the methylene protons of $CH_2NMe_2$. In contrast, the methyl part of $CH_2NMe_2$ consists of one singlet. The deshielding observed for one proton of the methylene group of $CH_2NMe_2$ is probably due to an agostic bond involving the gold atom. Interactions of this type have already been mentioned in the literature[13, 14] for other electrophilic metals and our finding seems to be one of the first examples reported for gold[15].

The very large deshielding of the $^{31}P$ signal for a bimetallic complex compared to the corresponding Au- free one is evidence for the coordination to gold.

$^{197}Au$ Mössbauer data for **4**, **7** and **8** are in good agreement with those for linear gold(I) compounds such as $[dppf(AuCl)_2]$[16] and $[Ph_3PAuCl]$[17,18]. The values observed for QS and IS by reference to gold metal lie in the ranges 7.47 - 7.71 mm $s^{-1}$ and 4.19 - 4.48 mm $s^{-1}$ respectively.

**Scheme 2.**

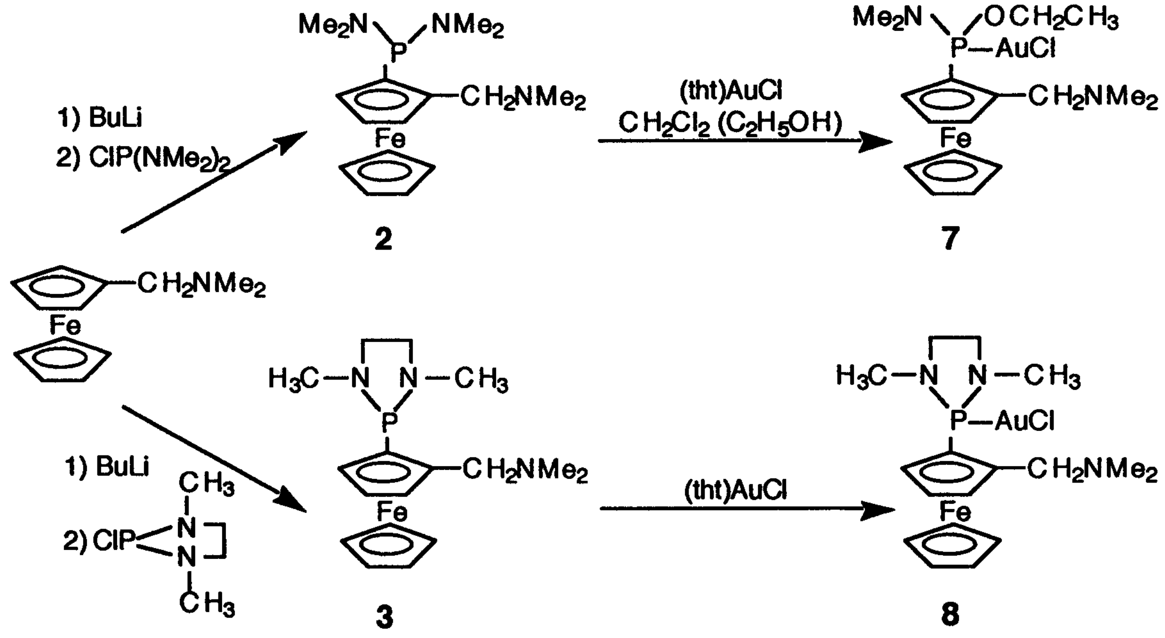

**Structural Studies**

The structure of compounds [Fc($CH_2NMe_2$)($PPh_2$)AuCl] (**4**) and [Fc($CH_2NMe_2$)(P($NMe_2$)($OCH_2CH_3$)(AuCl)] (**4**) were determined by X-ray diffraction.

**[Fc($CH_2NMe_2$)($PPh_2$AuCl)] (4)**

Figure 1 shows an ORTEP plot of one of the two enantiomers and Table IV contains selected bond lengths and bond angles. The cyclopentadienyl rings are eclipsed and parallel. The phosphorus and nitrogen atoms deviate from the best plane of substituted C1-C5 ring towards the iron atom by 0.15 and 1.13 Å, respectively, while the C6 and gold atoms deviate from this plane by 0.03 and 1.09 Å to the other side with respect to the iron atom. The nitrogen atom has a pyramidal geometry. The positions of methyl groups (C7 and C8) indicate the orientation of the nitrogen electron lone pair in the direction of gold, which might suggest an intramolecular Au-N interaction. However, such an interaction is excluded on the basis of extended Hückel MO calculations which show a slightly negative value of Au-N overlap. Thus, the gold atom is not perturbed by secondary interaction and exhibits an essentially linear geometry (Table IV). The Au-P and Au-Cl distances are close to the values observed in compounds with P-Au-Cl linkage[16-19].

**Figure 1.** ORTEP drawing of [Fc($CH_2NMe_2$)($PPh_2$AuCl)] (**4**)

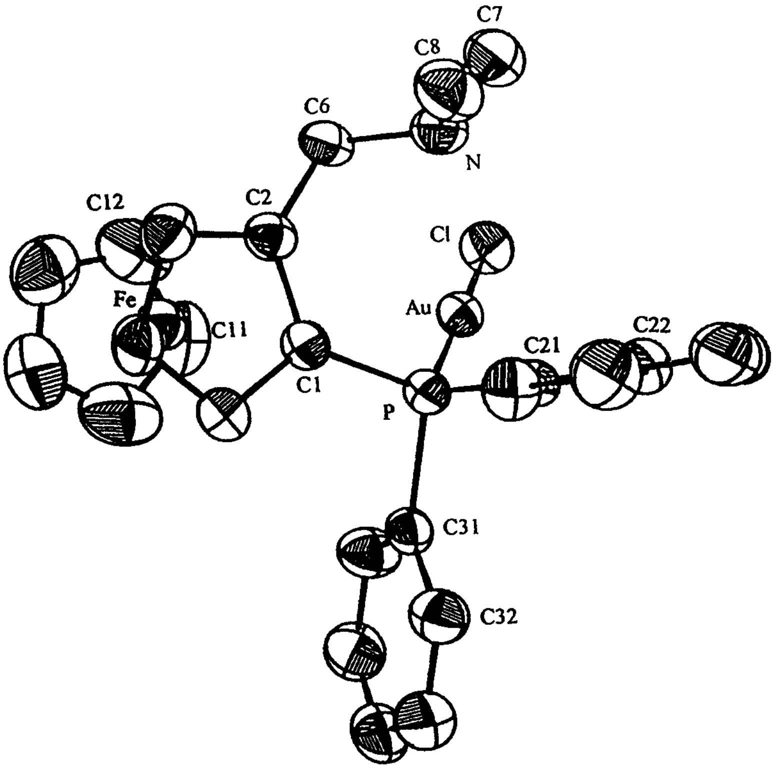

**Table IV.** Selected Interatomic Distances (Å) and Angles (°) for $[Fc(CH_2NMe_2)(PPh_2AuCl)]$ (**4**)

| | | | |
|---|---|---|---|
| Au...N | 3.53(1) | P-C1 | 1.79(1) |
| Au...Fe | 4.322(2) | N-C6 | 1.48(2) |
| Au-Cl | 2.276(3) | N-C7 | 1.44(3) |
| Au-P | 2.222(3) | N-C8 | 1.46(2) |
| P-C21 | 1.81(1) | C2-C6 | 1.50(2) |
| P-C31 | 1.83(1) | | |
| Cl-Au-P | 178.5(1) | N-C6-C2 | 111.6(9) |
| Au-P-C1 | 114.3(3) | C6-N-C7 | 111(1) |
| Au-P-C21 | 115.8(4) | C6-N-C8 | 111(1) |
| Au-P-C31 | 110.5(4) | C7-N-C8 | 111(1) |
| C1-C2-C6 | 127(1) | | |

**$[Fc(CH_2NMe_2)(P(NMe_2)(OCH_2CH_3)(AuCl)]$ (7)**

The presence of the ethoxy group has been confirmed by X-ray diffraction. A perspective drawing of one of the four isomers is represented in Figure 2. Selected bond lengths and angles are given in Table V. The cyclopentadienyl rings are essentially eclipsed and parallel. Contrary to the structure of **4**, the phosphorus and nitrogen atoms deviate (0.10 and 1.21 Å, respectively) from the best plane of C6-C10 ring to the opposite side with respect to the iron atom, while the C11 and Au atoms (deviations of 0.06 and 1.11 Å, respectively) are located between the planes of the Cp rings. Similarly to the structure of **4**, the nitrogen atom of the $CH_2NMe_2$ substituent (N1) has a pyramidal geometry, while the N2 bound to the phosphorus is planar. The sum of the three angles around N2 atom is equal to 359.9(9)°. This indicates the presence of $d\pi(P)$-$p\pi(N2)$ interaction. The P-N2 bond length of 1.640(9)Å is effectively intermediate between the values observed for phosphorus-nitrogen single and double bond[20]. On the other hand the P-O distance of 1.605(6)Å corresponds very well to the P-O single bond distance observed in P-O-C organic and inorganic linkages [20,21] . Even if the lone pair of the N1 atom roughly heads for the gold atom, the Au-N1 distance of 3.70(1)Å is longer than in **4**. Thus, we did not look for the existence of Au-N1 interaction. As expected, the P-Au-Cl bonding is linear.

**Figure 2.** ORTEPdrawing of $[Fc(CH_2NMe_2)(P(NMe_2)(OCH_2CH_3)(AuCl)]$ (**7**)

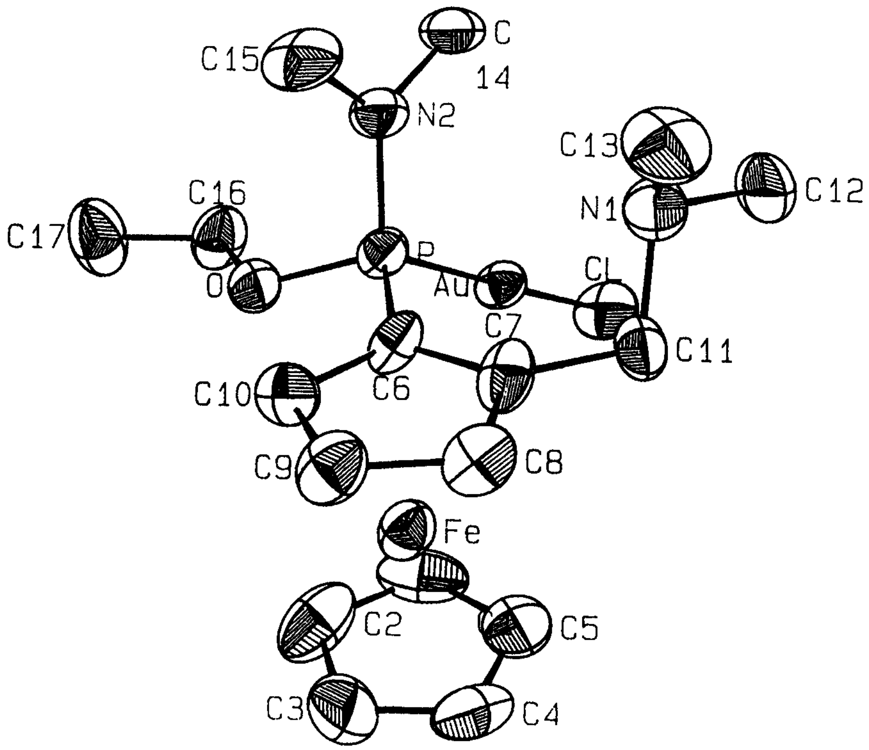

**Table V.** Selected Interatomic Distances (Å) and Angles (°) for $[Fc(CH_2NMe_2)(P(NMe_2)(OCH_2CH_3)(AuCl)]$ (**7**)

| | | | |
|---|---|---|---|
| Au...N1 | 3.70(1) | P - O | 1.605(6) |
| Au...N2 | 3.22(1) | P - N2 | 1.640(9) |
| Au...Fe | 4.374(1) | P - C6 | 1.797(9) |
| Au...O | 3.21(1) | N1 - C11 | 1.47(1) |
| Au - Cl | 2.303(3) | N1 - C12 | 1.44(2) |
| Au - P | 2.220(2) | N1 - C13 | 1.42(2) |
| N2 - C14 | 1.48(3) | N2 - C15 | 1.48(2) |
| ClAuP | 178.7(1) | OPC6 | 97.7(4) |
| AuPO | 114.1(2) | N2PC6 | 107.9(4) |
| AuPN2 | 111.1(3) | PN2C14 | 125.1(7) |
| AuPC6 | 117.9(3) | PN2C15 | 122.3(9) |
| OPN2 | 106.9(4) | C14N2C15 | 112.5(9) |

**Anti-tumour Testing**

The compounds were evaluated for potential anti-tumour activity using an *in vitro* screen composed of two human tumour cell lines, SW620 and HT1376. This approach has been adopted as *in vivo* screens for anti-tumour agents using transplantable murine tumours such as the L1210 and P388 leukaemias have had limited success in identifying drugs with activity against human solid tumours[22]. The two cell lines exhibit differential chemosensitivity towards the metal anti-tumour drug cisplatin with typical $IC_{50}$ values of 9.3 ± 1.5 $\mu$g ml$^{-1}$ for HT1376 and 29.5 ± 3.1 $\mu$g ml$^{-1}$ for SW620 (errors represent standard error of the mean, n = 9). Differential cytotoxicity is used as an indicator of anti-tumour activity whereas similar toxicity against both cell lines is taken as an indicator of non-specific cytotoxicity[23] The ratio of the highest $IC_{50}$ to the lowest $IC_{50}$ is a useful measure of differential cytotoxicity, this ratio is 3.2 for cisplatin.

The results for seven compounds are shown in Table VI.

**Table VI.** $IC_{50}$ ($\mu$g.ml$^{-1}$)

| Compound | Cell line | HT1376 (Bladder) | SW620 (Colon) |
|---|---|---|---|
|  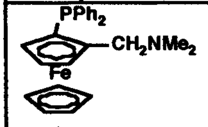 1 | | 6.9 | 16.5 |
|  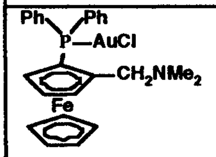 4 | | 9.7 | 5.4 |
|  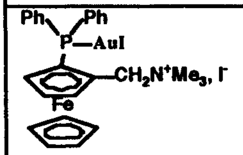 5 | | 16.2 | 73.0 |
|  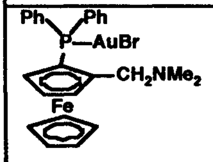 6 | | 1.4 | 10.2 |

| Compound | Cell line | HT1376 (Bladder) | SW620 (Colon) |
|---|---|---|---|
|  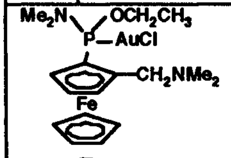 7 | | 1.5 | 7.4 |
|  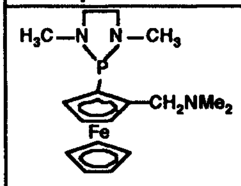 3 | | inactif | > 200 |
|  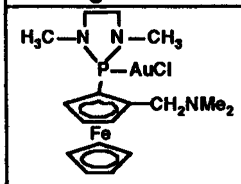 8 | | 1.3 | 19.6 |

Coordination of gold to compound **1** to give compound **4** results in a slight overall increase in cytotoxicity with an apparent inversion of the differential cytotoxicity in favour of the SW620 cell line. The differential however is not as great as that seen with cisplatin (ratio of highest $IC_{50}$ : lowest $IC_{50}$ is 2.4 for compound **1** and 1.8 for compound **4**). Compound **6**, the bromo analogue of compound **4**, shows both an enhanced cytotoxicity and differential activity (ratio of highest $IC_{50}$ : lowest $IC_{50}$ is 7.3) compared with compound **1**. The dimethylamino methyliodide salt, compound **5**, is less cytotoxic than compound **1** but with an enhanced differential cytotoxicity (ratio of highest $IC_{50}$ : lowest $IC_{50}$ is 4.5). The reduction in cytotoxicity is probably due to this being a charged compound, whereas the other complexes are neutral. This would reduce its ability to cross cell membranes and reach a putative intracellular target.

Complex **3**, the diazaphospholanyl ferrocene, is not cytotoxic, unlike the diphenylphosphino ferrocene, compound **1**. Interestingly, when coordinated to give the chlorogold complex, compound **8**, there is a dramatic increase in cytotoxicity. This is associated with an increase in differential activity with an $IC_{50}$ of 1.3 $\mu g\ ml^{-1}$ for HT1376 and 19.6 $\mu g\ ml^{-1}$ for SW620 giving a ratio of highest $IC_{50}$ : lowest $IC_{50}$ of 19.1. This compound is also more toxic towards the HT1376 cell line than compound **4**, the chlorogold diphenylphosphine. Compound **7**, the chlorogold dimethylaminoethoxyphosphine, is similarly cytotoxic towards the HT1376 cell line with a ratio of highest $IC_{50}$ : lowest $IC_{50}$ of 4.9.

These data indicate that the potency and differential cytotoxicity of phosphinogold(I) ferrocenes can be changed by chemical modification.

### ACKNOWLEDGEMENTS

We gratefully acknowledge Johnson Matthey Technology Centre (Reading-UK) for the loan of gold salts, Dr. Parish and the Microanalysis Centre in UMIST (Manchester-UK), the french "Ministère des Affaires Etrangères" for the award of a research student ship (M.V.), Mrs S. Gourier (U.B.) and Mr. G.R. Henderson ( J.M.) for their technical assistance.

### REFERENCES

1 C.K. Mirabelli, D.T. Hill, L.F. Faucette, F.L. McCabe, G.R. Girard, D.B. Bryan, B.M. Sutton, J.O. Bartus, S.T. Crooke, R.K. Johnson, *J. Med. Chem.*, **1987**, *30*, 2181.

2 C.K. Mirabelli, B.D. Jensen, M.R. Mattern, C.-M. Sung, S.-M. Mong, D.T. Hill, S.W. Dean, P.S. Schein, R.K. Johnson, S.T. Crooke, Anti-Cancer Drug Des. **1986**, *1*, 223.

3 A. Houlton, R.M.G. Roberts, J. Silver, *J. Organomet. Chem.*, **1991**, *418*, 107.

4 D. Lednicer, C.R. Hauser, *Org. Syntheses*, **1960**, *40*, 31.

5 D.W. Slocum, B.W. Rockett, C.R. Hauser, *J. Am. Chem. Soc.*, **1965**, *87*, 1241.

6 G. Marr, T. Hunt, *J. Chem. Soc. (c)*, **1969**, 1070.

7 R. Usón, A. Laguna, *Organomet. Syntheses*, **1986**, *3*, 322.

8 O. Bayer, H. Meerwein, K. Ziegler, in "Methoden der organischen Chemie", Georg Thieme Verlag, Stuttgart, Vol. 12, No. 2, **1964**, p. 105 et 108.

9 A. Leibovitz, J.C. Stinson, W.B. Mc Combs III, C.E. Mc Coy, K.C. Mazur, N.D. Mabry, *Cancer Res*, **1976**, *30*, 4562.

10 S. Rasheed, M.B. Gardner, R.W. Rongey, W.A. Nelson-Rees, P. Arnstein, *J. Natl. Cancer Inst*, **1977**, *58*, 881.

11 J.D. Higgins III, L. Neely, S.P. Fricker, *J. Inorg. Biochem.*, **1993**, *49*, 149.

12 I.E. Nifant'ev, A.A. Boricenko, L.F. Manzhukova, E.E. Nifant'ev, *Phosphorus, Sulfur and Silicon*, **1992**, *68*, 99.

13 M. Brookhart, M.L.H. Green, *J. Organomet. Chem.*, **1983**, *250*, 395.

14 A. Albinati, P.S. Pregosin, F. Wombacher, *Inorg. Chem.*, **1990**, *29*, 1812.

15 L.G. Kuz'mina, T.V. Baukova, N.A. Oleinikova, D.A. Lemenovskii, XVIth I.C.O.M.C. Communication OA. 19, Brighton, 1994.

16 Hill, D. T.; Girard, G. R.; McCabe, F. L.; Johnson, R. K.; Stupik, P. D.; Zhang, J. H.; Reiff, W. M.; Eggleston, D. S. *Inorg. Chem.*, **1989**, *28*, 3529.

17 Parish, R. V. in "Mössbauer Spectroscopy Applied to Inorganic Chemistry", G. J. Long ed., Plenum Press, N Y, Vol. 1, 1984, p. 577.

18 Al-Sa'ady, A. K. H.; McAuliffe, C. A.; Moss, K.; Parish, R. V.; Fields, R. *J. Chem. Soc., Dalton Trans.*, **1984**, 491.

19 H. Gornitzka, S. Besser, R. Herbst-Irmer, U. Kilimann, F.T. Edelmann, K. Jacob, *J. Organomet. Chem.*, **1992**, *437*, 299.

20 F.H. Allen, O. Kennard, D.G. Watson, L. Brammer, A.G. Orpen, R. Taylor, *J. Chem. Soc, Perkin Trans. II*, **1987**, S1.

21 M.M. Kubicki, W. Wojciechowski, *J. Mol. Struc.*, **1976**, *33*, 201.

22 T.H. Corbett, F.A. Valeriote, L.H. Baker, *Invest New Drugs*, **1987**, *5*, 3.

23 S.P. Fricker, *Metals Ions in Biology and Medicine*, eds P. Collery, L.A. Poirier, M. Manfait, J.C. Etienne, John Libbey Eurotext, Paris, **1990**, p. 452.

**Received: June 8, 1995 - Accepted: July 14, 1995 - Received in revised camera-format: November 10, 1995**

